# Combining Spatial Analysis and a Drinking Water Quality Index to Evaluate Monitoring Data

**DOI:** 10.3390/ijerph16030357

**Published:** 2019-01-27

**Authors:** Hongxing Li, Charlotte D. Smith, Li Wang, Zheng Li, Chuanlong Xiong, Rong Zhang

**Affiliations:** 1 National Center for Rural Water Supply Technical Guidance, Chinese Center for Disease Control and Prevention, Beijing 102200, China; lihongxing@crwstc.org or lihx@ncrwstg.chinacdc.cn (H.L.); wangli@ncrwstg.chinacdc.cn (L.W.); lizheng@crwstc.org (Z.L.); xiongcl@ncrwstg.chinacdc.cn (C.X.); 2 Division of Environmental Health Sciences, School of Public Health, University of California, Berkeley, CA 94720, USA; charlottesmith@berkeley.edu

**Keywords:** drinking water, water quality index, spatial analyses, monitoring

## Abstract

Drinking water monitoring is essential for identifying health-related risks, as well as for building foundations for management of safe drinking water supplies. However, statistical analyses of drinking water quality monitoring data are challenging because of non-normal (skewed distributions) and missing values. Therefore, a new method combining a water quality index (WQI) with spatial analysis is introduced in this paper to fill the gap between data collection and data analysis. Water constituent concentrations in different seasons and from different water sources were compared based on WQIs. To generate a WQI map covering all of the study areas, predicted WQI values were created for locations in the study area based on spatial interpolation from nearby observed values. The accuracy value of predicted and measured values of our method was 0.99, indicating good predication performance. Overall, the results of this study indicate that this method will help fill the gap between the collection of large amounts of drinking water data and data analysis for drinking water monitoring and process control.

## 1. Introduction

Drinking water quality is an important public health concern because it is highly correlated with diseases such as cholera, typhoid, dysentery, legionellosis, and schistosomiasis. Accordingly, it is essential to manage drinking water quality from source to tap to protect the public from disease and maximize the health benefits of clean water such as adequate hydration and hygiene [1]. An understanding of regional drinking water quality is a prerequisite to drinking water management [2] and drinking water quality monitoring is a useful tool for the evaluation of regional water quality status [3]. Drinking water quality monitoring enables the identification of water quality problems, allowing corrective measures to be taken when necessary [4]. However, monitoring data can only be used for drinking water management or decision-making after appropriate data analysis has been completed [5]. Unfortunately, there is still a lack of suitable analyses and information reporting protocols for drinking water quality monitoring data, despite many years of world-wide monitoring experience.

Since water quality is not determined by a single parameter, it can be described based on a variety of water quality indices [6]. However, such results can be difficult to understand, especially for non-technical personnel. Therefore, a method of evaluating drinking water monitoring data based on the proportion of water samples in compliance with drinking water standards offers an alternative to constituent by constituent assessment and reporting. However, using this method to describe monitoring data also has limitations. For example, a summary value will hide specific water quality results, as well as the degree to which samples exceeded the standard. Water quality indices (WQI) are another way of describing water quality monitoring data in which a large number of variables are converted to a one- or two-digit number [7]. Such indices are useful in the monitoring and management of water quality because they can be used as a convenient tool to examine regional water quality trends [8], highlight specific environmental problems [9], and help decision-makers to evaluate the effects of specific water quality intervention measures [10].

Many studies have integrated WQIs and geographical information systems (GIS) to generate maps [11], assess ground water quality [12,13], drinking water infrastructure assessment [14], and describe spatial and temporal variations in water quality [15,16]. Since drinking water monitoring data always have time and space attributes, GIS is an excellent tool for displaying and analyzing monitoring data. Limited by resources, in a real drinking water quality monitoring program, one cannot cover every water supply point. Hence, when making a drinking water quality map or analyzing water quality trends, it is necessary to do some interpolation based on existing data [17,18]. In this study, we combined WQIs and a spatial interpolation method to describe regional drinking water quality trends. The results presented here will contribute to a more comprehensive method for drinking water quality monitoring data analysis. The results, which are easily visualized, can then be the helpful for safe drinking water supply operation and management.

## 2. Materials and Methods 

### 2.1. A Regional Drinking Water Quality Monitoring Plan

**Figure 1:**
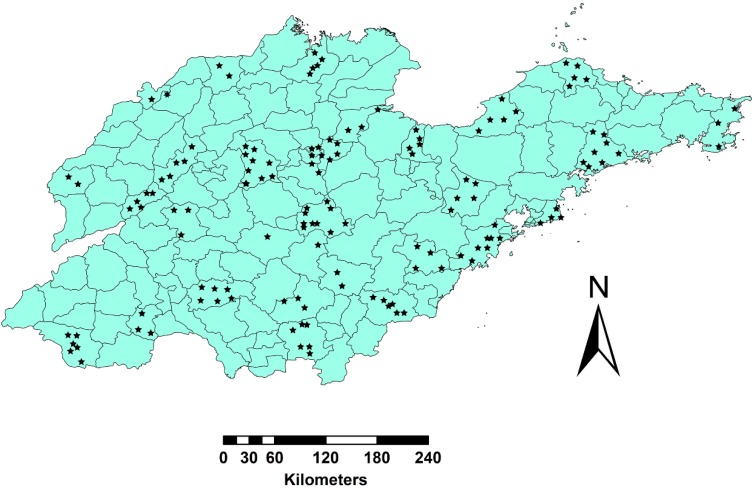
Distribution of water quality monitoring points in Shandong province.

The data used in this study were from a historical drinking water quality monitoring program in the study region from 2007 to 2011 in Shandong province, China. Since the study focused on a drinking water quality data analysis method and drinking water quality problems are a sensitive topic, detailed information regarding the study area were omitted. Overall, 128 of 1120 total water supply points were selected for analysis. These water supply points were selected to encompass different (sources, treatment types, and geographical areas). The distribution of the 128 the water quality monitoring points in the study areas are shown Figure 1.

These 128 drinking water quality monitoring points covered both centralized water systems and decentralized water systems, such as hand pumps and dug wells. The detailed information about these 128 drinking water quality monitoring points are provided in Table A1. The proportion of different sources, and treatment levels were selected to create a representative sample of the province. Table A2 lists the methods for each water quality parameter. From 2007 to 2011, we collected treatment plant finished water and distribution system (tap water) for the centralized system, as well as domestic use water for the decentralized systems. The water samples were tested at laboratories of the Disease Control and Prevention Centers, and sampling and testing were repeated in dry and wet seasons each year. Basic information (e.g., source, location, date, time) was collected at the time of sample collection. In this drinking water quality monitoring program, 16 water quality indicators were collected including color, turbidity, odor, pH, total hardness, iron, manganese, sulfate, chloride, total dissolved solids, chemical oxygen demand (COD), fluoride, arsenic, nitrate, total coliform bacteria (TC), and thermotolerant coliform bacteria (TCB).

The following WQI was computed by the addition of unweighted sub-indices:

(1) $$I=\sum_{i=1}^{n}I_{i}$$

where *I_i_* is the sub-index for water quality parameter *I* and *n* is the number of total water quality parameters.

(2) $$I_{i}=C_{i}/S_{i}$$

(3) $$I_{pH}=\left(C_{pH}-7\right)/1.5$$

(4) $$I_{TC}=\{\begin{array}{cc} 0, & C_{i}=0\\ n\left(C_{i}+1\right)+1, & C_{i}>0 \end{array}$$

(5) $$I_{TCB}=\{\begin{array}{cc} 0, & C_{i}=0\\ n\left(C_{i}+1\right)+1, & C_{i}>0 \end{array}$$

The sub-indices were calculated using Equations (2–5). Equation (1) was used to create a single index value for the results of the following constituents: color, turbidity, odor, total hardness, iron, manganese, sulfate, chloride, total dissolved solids, oxygen demand, fluoride, arsenic, and nitrate. *I_i_* is the sub-index of the organoleptic indicator, *C_i_* is the measured concentration of indicator *I*, and *S_i_* is the value of indicator *I* in China’s national drinking quality standards. *I_pH_*, which is calculated using Equation (3), is the sub-index for pH and *C_pH_* is the pH value of the drinking water. Equations (4) and (5) were used to calculate the two microbiological indicators (TC and TCB).

### 2.2. Spatial Temporal Assessment for WQIs

Geographic Information Systems have been widely applied for complex modeling in water related studies. To create a WQI map covering all of the areas investigated in this study, predicted WQI values were created for locations in the research region based on spatial interpolation from nearby observed values. Spatial interpolation is a statistical technique that estimates the value of all unknown points between known points [19]. Kriging is the most commonly used interpolation method [20]. There are several different kriging methods, for example: ordinary kriging, simple kriging, universal kriging, disjunctive kriging, and empirical Bayesian kriging [21,22]. Ordinary kriging, which is the most common and widely used kriging method, was employed in this study.

In this paper, cross-validation [23] was used to verify the accuracy of the interpolation results. Statistical indicators that indicate the degree of concordance between the models and reality (mean absolute error (MAE), mean relative error (MRE), and the root mean square error (RMSE)) were calculated [24]. The MRE represents the percentage of error between the observed and predicted WQI, while the RMSE and MAE summarize the mean differences between observed and predicted WQIs. The specific equations used were as follows [25]:

(6) $$MAE=\frac{1}{n}\sum_{i=1}^{n}\left|WQI_{oi}-WQI_{ei}\right|$$

(7) $$MRE=\frac{1}{n}\sum_{i=1}^{n}\left|\frac{WQI_{oi}-WQI_{ei}}{WQI_{oi}}\right|$$

(8) $$RMSE=\sqrt{\frac{1}{n}{\sum_{i=1}^{n}\left(WQI_{oi}-WQI_{ei}\right)}^{2}}$$

where *n* is the number of water sample points and *WQI_oi_* and *WQI_ei_* are the measured and predicted WQI values, respectively. Based on these indicators, the accuracy (AC) of predicted and measured values could be calculated using Equation (9):

(9) $$AC=1-\frac{nRMSE^{2}}{\sum_{j=1}^{n}{\left[\left|WQI_{oi}-WQI_{oe}\right|+\left|WQI_{ei}-WQI_{oe}\right|\right]}^{2}}$$

where *M* is the mean measured *WQI_oe_* value. The accuracy, calculated using Equation (9), varied between 0 and 1, with larger values indicating better predicted results.

### 2.3. Quality Control and Data Management

The collection, transportation, preservation, and detection of water samples were carried out in accordance with national standard GB 4750–2006 [26]. The basic information of 128 points and water quality test results were reported through a web-based information system, and the outlier data records were double-checked. We used the R software program to extrapolate all of the WQIs in the study areas based on regional maps. R packages gstat and ggplot2 were used for spatial analysis and visualization [27].

## 3. Results

### 3.1. Basic Information Regarding 128 Drinking Water Quality Monitoring Points

Drinking water quality data from 128 monitoring points based on drinking water monitoring networks were collected in 2007–2011 according to the monitoring plan. Since the water quality testing was performed in both the dry season and the wet season each year, the total number of observations was 1280. The total number of water samples classified by water sources, water supply type (centralized or decentralized) and season (dry or wet) are shown in Table 1.

**Figure 2:**
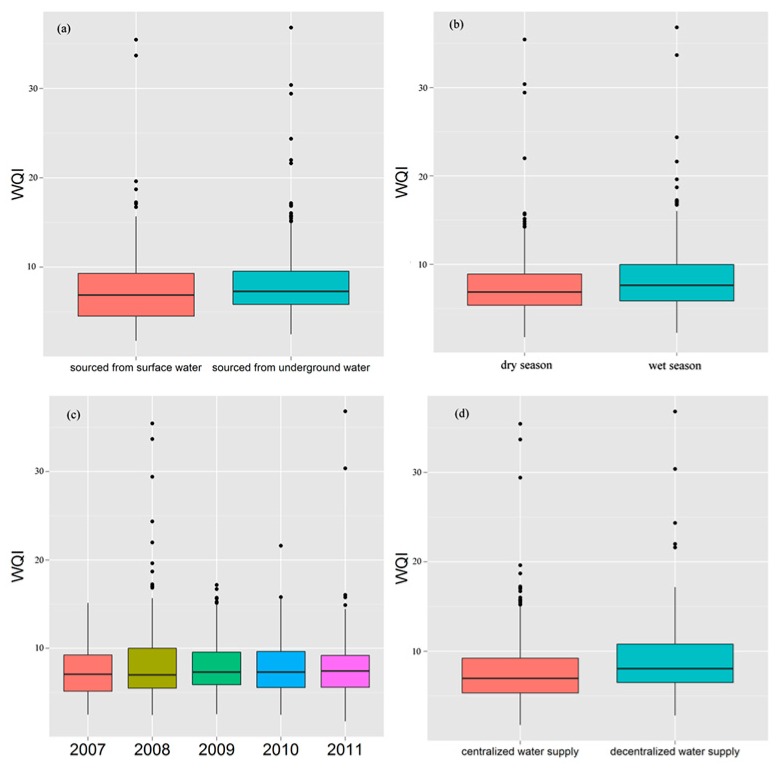
Boxplot of water quality indices (WQIs) of various drinking water samples.

Figure 2 shows the distribution of WQIs in different water supply types, seasons, years and water sources, which provided a direct visualization of water quality. A two-sample *t*-test was conducted to compare the WQIs associated with different water supply types, seasons and water sources, and ANOVA was used to compare the WQIs in different years. The drinking water quality from ground water was better than that of surface water (*p* < 0.05), and there was no difference in water collected in the dry season relative to the rainy season (*p* = 0.378). The quality of the centralized water supply was better than that of the decentralized water supply (*p* < 0.01). The quality of drinking water in different years was also compared using a similar method. The WQIs in 2008 were significantly higher than that in other years (*p* < 0.05).

### 3.2. Spatial and Temporal Distribution of WQIs

**Figure 3:**
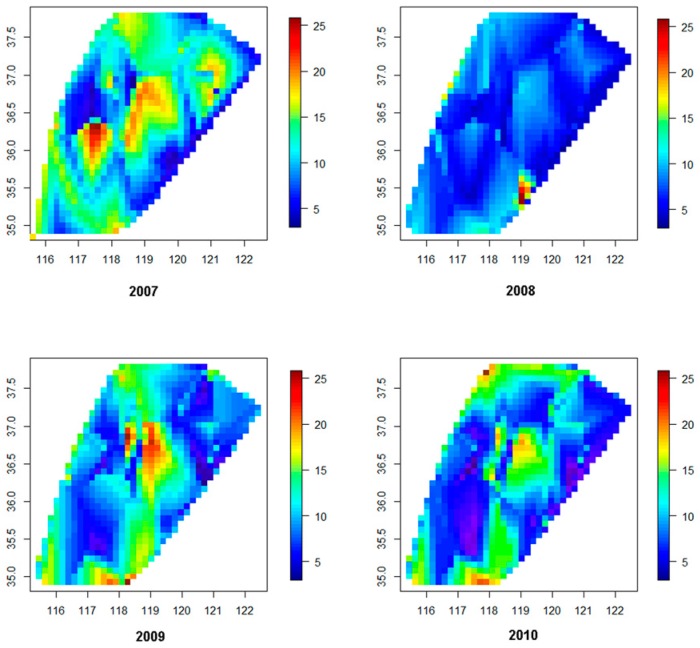
Temporal distributions of WQIs in the study area.

The spatial and temporal distribution of the WQIs was also investigated using the ordinary kriging method. In Figure 3, the horizontal axis indicates longitude, the vertical axis indicates latitude, blue areas indicate small WQIs, and red areas indicate high WQIs. Hence, we could evaluate the spatial distribution of WQIs from the distribution of the colors. The graphs provide a visual representation of the variations in water quality in time and space. As shown in Figure 3, the drinking water quality in the study area was better in 2008 than in 2007, 2009, and 2010, (as there were more blue areas in the 2008 map). Furthermore, drinking water quality of the central region was far worse than that of other areas as indicated by the distribution of the red areas. In summary, this type of spatial map can be used to describe the spatial and temporal distribution of drinking water quality indices.

### 3.3. Interpolation Performance

The MAE, MRE, and RMSE of the interpolation model were 0.0034, 0.0027, and 0.085, respectively, which are all very low and indicate good fit of the model to actual data. Additionally, the AC value of this spatial model was 0.994, which indicates good performance.

## 4. Discussion

Drinking water safety, especially in rural areas, is of great concern to governments and non-governmental organizations, as drinking water safety is closely related to socioeconomic development [28]. Ensuring access to clean water and safe sanitation for all are among the most important sustainable development goals [29]. Therefore, the United Nations has developed a standardized module for drinking water quality testing in close collaboration with the Multiple Indicator Cluster Survey (MICS) program [30]. Drinking water quality monitoring covering water supply systems, such as various treatment stages, distribution networks, and households has also been conducted in many countries [31]. These types of monitoring programs are essential to the identification of problems and appropriate interventions to protect public health [32]. Significant advances have been made in the collection of water quality data, real-time management of data, and communications [33]. This progress means that a greater amount of water quality data is currently collected and there is a higher demand for data analysis and visualization methodologies.

Although drinking water quality data are important for public health departments and water supply agencies, few studies have evaluated drinking water quality data analysis methods. In general, much more water quality data is collected relative to the amount of data analysis conducted [34]. There are several possible reasons for this discrepancy: (1) statistical analyses of drinking water quality data are still challenging because of non-normal distributions and missing values [35,36]; (2) for time-based or seasonal models, the frequency is often not sufficient to overcome variability and uncertainty; and (3) it is difficult to summarize the time-space dimensions of water quality data.

In this study, we developed a method for combining spatial analysis with WQIs for drinking water quality data. Since the WQI was the first index used to evaluate water quality [37], many researchers have developed different WQIs for various purposes, including the weighted sum index, geometric mean index, maximum operator index, minimal operator index, and Nemerow index [6]. However, those indices do not simultaneously incorporate temporal and spatial components of the data. As comprehensive water quality assessment tools, WQIs can reduce the dimensions of water quality indices to a single index, simplifying the comparison of water samples. WQIs used in this paper give a direct and visual result showing that water quality from public water supplies was better than the decentralized water systems (primarily well water). This difference in water quality may be attributed to stricter management and better treatment of public water supplies. The drinking water quality difference from ground water and surface water could be attributed to the fact that groundwater is protected from microbial contamination due to sub-surface filtration (through sand and soil). There was no difference for drinking water quality in the wet and dry seasons, as the deep groundwater was the main drinking water source in Shandong province, which was less affected by season. The results also showed the annual variation of drinking water quality, which indicate better water quality in 2008. This may be attributed to better source water quality or stricter water quality management measures in 2008. When compared with other water quality evaluation tools, WQIs can reflect the quality of water more exactly. At the same time, this method can help establish health-based targets by regulatory agencies. Water quality indices can also be used by water suppliers when assessing their treatment plant process control to ensure drinking water safety especially during short-term events when fluctuations of drinking water quality may be significant.

The innovation of this study lies in the fact that we combined the WQI with spatial analysis. The potential values of this method are: (1) Such a WQI map could directly reflect the status and the variation of the drinking water quality, especially for the general public. (2) This method could be helpful in finding regional water quality risk points and provide a basis for water quality risk management. A systemic drinking water management tool known as water safety plans (WSP) was recommended by WHO [4]. When combined with WSP, our method could be useful to find weak points or risk points in a region or in a water supply system. (3) Our method provided a methodological basis for epidemiological research on water quality and health. Synthesis and extrapolation of water quality information is often needed in epidemiological studies on the relationship of water quality to diseases.

## 5. Conclusions

We have developed a method for combining water quality indices and spatial interpolation to analyze and visualize regional drinking water quality data. This method will help fill the gap between the large amount of drinking water monitoring data collected and the need to analyze these data. In this study, we applied spatial methods to evaluate the WQIs over the entire study area. Since water and health data have spatial attributes [38], it is useful to visualize these data on a GIS-produced map. Combining the WQIs with GIS enables more in-depth analysis of drinking water quality and therefore is a positive contribution to the protection of public health.

## Figures and Tables

**Table 1:** Number of drinking water samples from monitoring points.

Year	Sources		Water Supply Types		Seasons	
	Groundwater	Surface Water	Centralized	Decentralized	Dry	Wet
2007	182	74	182	74	128	128
2008	208	48	196	60	128	128
2009	204	52	206	50	128	128
2010	202	54	208	48	128	128
2011	202	54	208	48	128	128

## Appendix A

**Table A1:** Detailed information on the 128 points in 2011.

Sources		Public Water Supplies			Decentralized Water Supplies	
		Treated *	Partially Treated **	Untreated ***	Hand Pump	Dug Well
Surface water:	river	1	1	-	0	1
	reservoir	21	5	-	0	0
Groundwater:	well	-	22	54	1	21
	spring	-	0	1	0	0

* Treated means conventional treatment, (coagulation, flocculation, sedimentation, filtration, and disinfection); ** Partially Treated means direct filtration (flocculation and sedimentation absent) for surface or disinfection only for Ground water; *** Untreated means no treatment.

**Table A2:** Methods for water quality parameter.

Parameter	Method	Parameter	Method
Color	Eye-measurement colorimetry of platinum-cobalt color-code	Chloride	Silver nitrate volumetric method
Turbidity	Scattering method (Formazine standard)	Total dissolved solids	Weighing method
Odor	Smell method	Chemical oxygen demand (COD)	Acidic Potassium Permanganate Titration
pH	Glass electrode method	Fluoride	Ion-selective electrode method
Total hardness	EDTA titration method	Arsenic	hydride atomic fluorescence method
Iron	Atomic Absorption Spectrophotometry	Nitrate	Ultraviolet spectrophotometry
Manganese	Atomic Absorption Spectrophotometry	Total coliform bacteria (TC)	Plate counting method
Sulfate	Combustion-barium sulfate turbidimetry	Thermotolerant coliform bacteria (TCB)	Multi-tube Fermentation Method
